# Changing trends in child welfare inequalities in Northern Ireland

**DOI:** 10.23889/ijpds.v8i2.2351

**Published:** 2023-09-14

**Authors:** Lisa Bunting, Nicole Gleghorne, Aideen Maguire, Sarah McKenna, Dermot O'Reilly

**Affiliations:** 1 Queen's University, Belfast, United Kingdom

## Objectives

This study uses longitudinal administrative data to investigate the relationship between area level deprivation and the 1) referral, 2) investigation, 3) registration and 4) looked-after stages of children’s contact with child and family social work in Northern Ireland (NI) from 2010-2017 (stages 1-3) and 2010-2020 (stage 4).

## Methods

Children’s social care data (SOSCARE database) for the years 2010 to 2020 were obtained from the Honest Broker Service in NI. The data were linked with the 2017 NI Multiple Deprivation Measure through the family of origin postcode. Cross-tabulations of year and deprivation decile were used to produce frequencies of children who experienced the four levels of intervention within each of the study years. These were then used to calculate various measures of absolute and relative inequality including the Slope Index of Inequality (SII), the Relative Ratio of Inequality (RRI) and the Relative Index of Inequality (RII).

## Results

There was a clear and increasing social gradient in child welfare interventions over time. Children referred to children’s social care during 2010-2017 were 4-5 times more likely to come from the most deprived areas compared to the least deprived. Despite fairly stable levels of referral inequality, the ratio of children subject to child protection investigations rose from 3 in 2010 to 6 in 2017, the ratio of children subject to child protection plans rose from 4.5 in 2010 to 8 in 2017 and the ratio of children looked after rose from 4 in 2010 to 9 in 2020. This widening inequality was largely driven by the increasing involvement of younger children from the most deprived areas in child protection and looked-after processes.

## Conclusion

In an environment of economic austerity and reduced spending, we are intervening in the lives of children and families living in the most deprived areas of NI at disproportionate rates. The current independent review of children’s social care offers an opportunity to reconfigure current provision with a clear inequalities focus.

